# Ventral versus dorsal onlay buccal mucosal graft urethroplasty for non-traumatic proximal bulbar urethral strictures in sexually active men: erectile and urinary functions

**DOI:** 10.1007/s00345-025-05441-7

**Published:** 2025-01-27

**Authors:** Ayman Mousa, Ahmed Eissa, Ali Abdel Raheem, Ahmed Zoeir

**Affiliations:** Urology Department, Faculty of Medicine, Tanta University, Tanta, Egypt

**Keywords:** Dorsal technique, Erectile functions, IIEF score, Proximal bulbar urethral strictures, Urethroplasty, Ventral technique

## Abstract

**Purpose:**

To compare the erectile and urinary functions of ventral and dorsal onlay buccal mucosal graft (BMG) urethroplasty in the management of proximal bulbar urethral strictures (PBUS) in sexually active men.

**Patients and methods:**

We retrospectively included patients with primary non-traumatic PBUS who were treated with (BMG) urethroplasty at our department between March 2019 and March 2023 either ventral or dorsal approaches. Patients were assessed at 3- and 12-months postoperatively for urinary and erectile functions.

**Results:**

133 patients were identified and underwent either ventral repair (*n* = 60, group I) or dorsal repair (*n* = 73, group II). There was no significant difference in baseline urinary and sexual function between both groups (*p* > 0.05). Overall, the success rate was 91.7% in Group I and 90.4% in Group II (*p* = 0.801). Transient ED (at 3 months) was detected in 5% and 25% (*p* = 0.002), while permeant ED (at 12 months) was 1.7% and 13.7% (*p* = 0.012) in group I and group II, respectively. Group I had significantly higher mean IIEF scores; 28.2 and 28.4 at 3 months (*p* < 0.001) and 12 months (*p* < 0.001); compared to Group II; 22.1 and 24.4, respectively.

**Conclusion:**

The ventral approach had better erectile functional outcomes compared to the dorsal approach in the management of non-traumatic PBUS in sexually active men. This might be related to less urethral mobilization and no dissection of the intercrural space during ventral onlay graft urethroplasty.

**Supplementary Information:**

The online version contains supplementary material available at 10.1007/s00345-025-05441-7.

## Introduction

Treatment of proximal bulbar urethral strictures (PBUS) in sexually active men is challenging because of the associated risk of sexual dysfunction resulting from the proximity of cavernous nerves and arteries [1]. Numerous surgical techniques have been proposed for treating bulbar urethral strictures, such as the use of free graft versus pedicled flap, ventral versus dorsal graft placement, non-transecting technique, etc. According to the literature, none of these techniques is superior to the others as they are all associated with similar success and complication rates. Thus, the selection of the appropriate technique remains challenging [1].

Despite anastomotic bulbar urethroplasty has a better success rate compared to urethral dilatation, its application has been limited due to its drawbacks on erectile function with 23.3%, 18.3%, and 1.6% of patients developed ejaculatory dysfunction, reduced glans sensitivity, respectively [2, 3]. The non-transecting anastomotic bulbar urethroplasty preserves the urethral and corpus spongiosum arteries with no reported sexual complications, however, the deep operative field of the proximal bulb makes it more challenging to adopt this procedure in PBUS [4]. Since the introduction of the buccal graft use for bulbar urethroplasty [5–7], the use of dorsal or ventral grafting is still a debatable issue although several studies reported a comparable success rate for the dorsal and ventral onlay techniques (80–95%) and (79-90%), respectively [8–11]. Nowadays, most surgeons prefer the ventral technique because it is associated with lower risk of post-urethroplasty erectile dysfunction (ED) and the the thick spongiosum gives good graft support [12].

Previous anatomical studies evaluated the anatomy and the relation of the membranous and bulbar urethra to the neurovascular bundles (NVB) showing that at the level of the proximal bulbar urethra, the majority of cavernous nerve fibers progressively move to the 1 and 11 o’clock locations at the point where the two crus of the corpora cavernosa assemble towards the midline distally. Accordingly, dissecting the intercrural space may be associated with increased risk of ED as it contains nerves related to the erection [13, 14]. A recent study evaluated the use of dorsal onlay urethroplasty in the management of membranous urethral stricture showing that approximately 70% of the histologically excised tissues from the intercrural space contained nerve tissues [15].

Accordingly, we hypothesized that dissecting the intercrural space between 1 and 11 o’clock locations at the level of the proximal bulbar urethra during the dorsal onlay technique may increase the risk of ED due to the possible risk of damage to the nerves and vessels crossing this area, as we need to go deeper and wider during the dissection. Additionally, the use of a ventral approach in PBUS is safer in young sexually active men to maintain their erection after urethroplasty as it needs less urethral mobilization and avoids dissection in the intercrural space.

To our knowledge, this is the first study in the literature that specifically focuses on managing PBUS and compares both techniques. Previous studies compared both techniques have included the whole bulbar urethra; however, the anatomy of the nerves and vessels is different at the level of the proximal, mid, and distal bulbar urethra. The current study aims to compare the urinary and erectile functions in both ventral and dorsal BMG urethroplasty in sexually active men.

## Materials and methods

### Study type and patients’ selection

After Local ethical committee approval was obtained (IRB protocol code: 36264PR626/4/24), we retrospectively revised the urethroplasty database at our hospital, to identify patients who underwent BMG urethroplasty between March 2019 and March 2023. Overall, 550 patients underwent urethroplasty during the study period, of which 137 patients were documented to have primary non-traumatic PBUS and underwent BMG urethroplasty either ventral or dorsal approaches. We excluded 2 patients with missed follow-up and 2 patients with missed main study outcomes’ data. At our center, initially, we used the dorsal onlay BMG urethroplasty *(DOBMGU*, *dorsal group)* for PBUS from 2019 to 2021, however, when we documented many cases of post-urethroplasty ED, we switched to the ventral technique *(VOBMGU*, *ventral group)* from 2021 to 2023.

We reviewed the retrograde urethrogram (RUG) images of all patients to document the stricture length and site (Fig. 1S in supplementary material 1). Sexually active men with primary non-traumatic PBUS were included. Patients with preoperative ED, pan-urethral strictures, traumatic strictures, and/or a history of previous urethroplasty were excluded.

### Preoperative patients’ evaluation

The patients were assessed by detailed history taking and thorough examination. The baseline maximum flow rate (Q-max) and IIEF score are routinely performed for all patients planned for urethroplasty at our center. Sexually active man in our study is defined as men who had weekly or more sexual activity in the past year [16]. We used the erection domain of IIEF-15 questionnaire which consists of (Q1,2,3,4,5, and 15). RUG was done for all patients to assess the stricture site and length.

### Operative techniques

Single-dose prophylactic antibiotic was given to all patients according to our hospital protocol. Under general or spinal anesthesia, the patient is positioned in a dorsal lithotomy position with proper padding of the pressure points. The perineum is prepared and sterilized by betadine and then, the surgical field is covered with sterile drapes. Buccal graft harvesting was done simultaneously by another team to save time and limit the infection risk. In patients undergoing spinal anesthesia, BMG was harvested under local infiltration anesthesia (Fig. 2S in supplementary material 1). Initially, we performed urethroscopy using a 6 F ureteroscope (*Richard Wolf*, *Germany*) to assess the stricture site and length and to introduce a Terumo guide wire into the bladder (Fig. 3S in supplementary material 1). A midline perineal incision is done in both groups after intra-urethral injection of 5–10 ml of methylene blue. All surgeries were performed by a single expert reconstructive surgeon.

#### Technique of VOBMGU

**Fig. 1:**
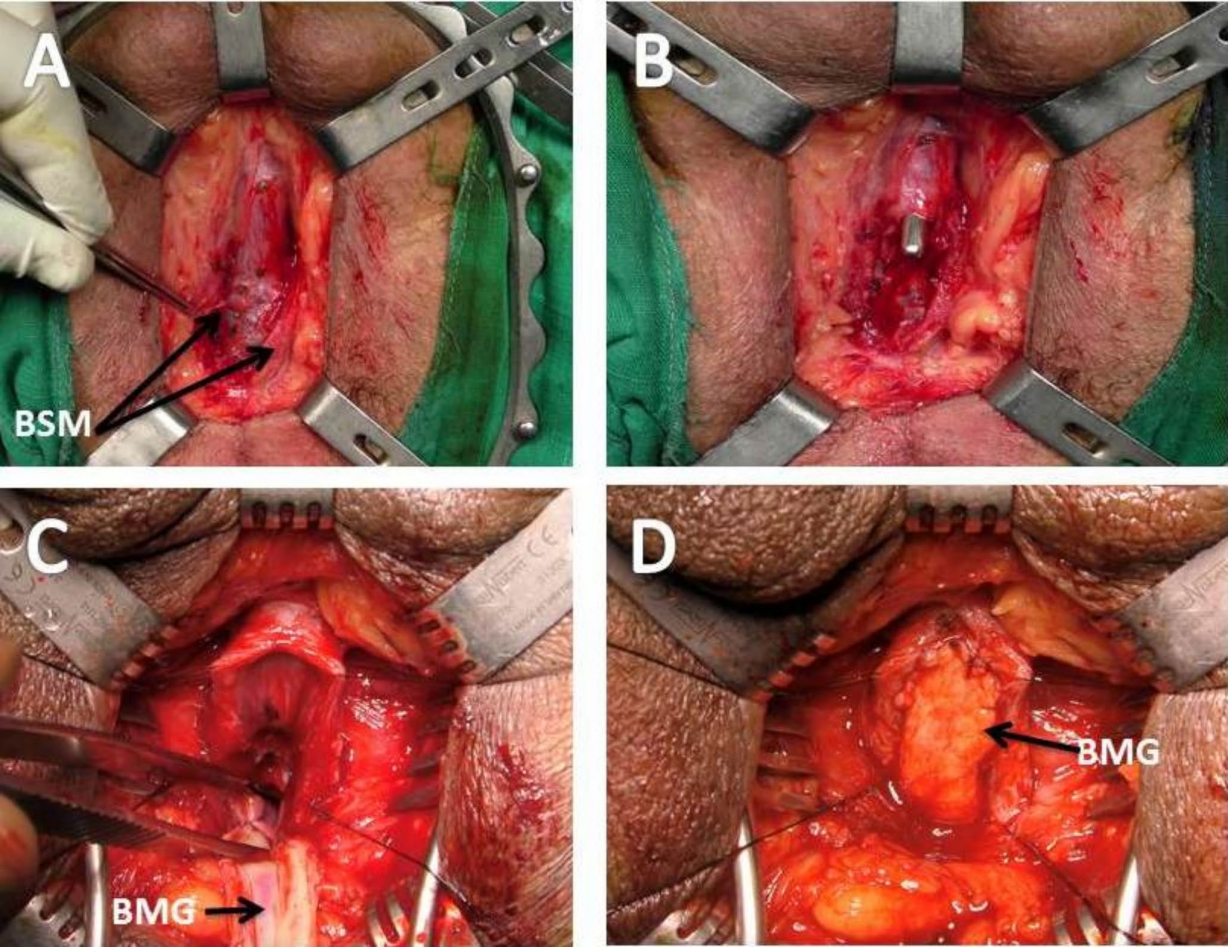
Operative steps of VOBMGU. **A** The bulbospongiosus muscle (BSM) is divided in the midline to expose the underlying bulb *(the black arrows)*. **B** Opening the urethra ventrally over a urethral dilator (16-F). **C** Proximal anastomosis of BMG to the urethra by three interrupted sutures. **D** Suturing of the graft to both urethral edges over a 16-F urethral catheter

The bulbospongiosus muscle (BSM) is cut in the midline to expose the bulb. The urethra is opened ventrally over a urethral dilator and at the most distal stricture site. The graft is anastomosed proximally to the urethra with three interrupted absorbable sutures (4 − 0 Vicryl) close to the verumontanum without involving it (Fig. 1). Then, the urethral edges are sutured to the graft and the spongiosum is closed over it, after the insertion of silicone urethral catheter (16 F).

#### Technique of DOBMGU

**Fig. 2:**
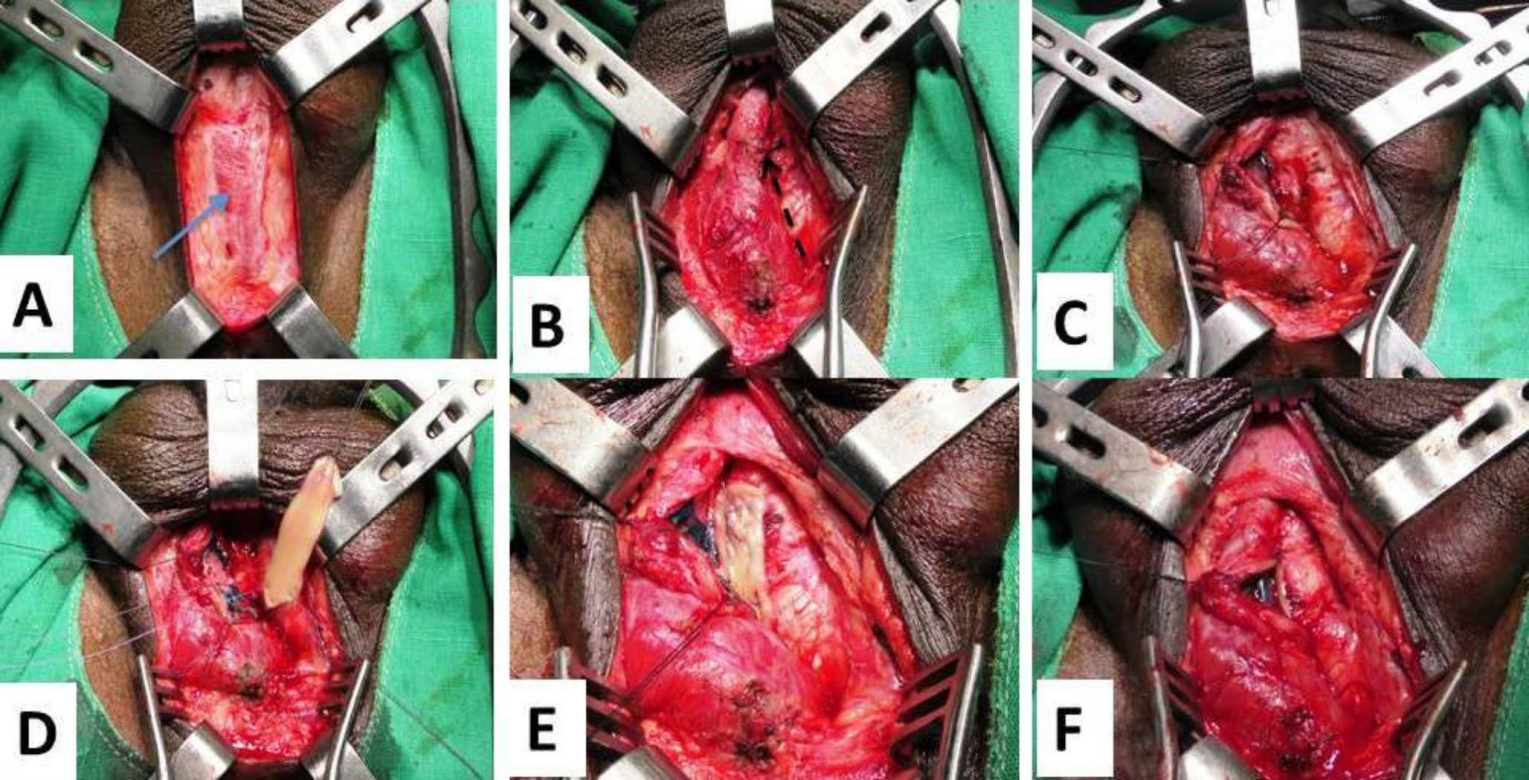
Operative steps of DOBMGU. **A** Midline perineal incision that exposes the underneath bulbospongiosus muscle *(the blue arrow)*. **B** One-sided dissection between the urethra and left corpora cavernosa *(the dotted black arrow)*. **C** Opening the urethra dorsally. **D** Proximal anastomosis of the BMG to the urethra by three interrupted sutures. **E** Quilting of the graft to the underlying corpora by three rows of sutures. **F** Completion of anastomosis of the graft to urethral edges over a 16-F urethral catheter

one-side dissection technique is performed to dissect and mobilize the bulbar urethra, and then the urethra is opened dorsally just distal to the stricture, going through the stricture with more dissection dorsally to pass the stricture to the normal membranous urethra. Then, the graft is anastomosed proximally to the urethra with 3 interrupted sutures (4 − 0 Vicryl). Quilting of the graft to the corpora cavernosa is done by three rows of sutures. Subsequently, the urethral edges are sutured to the graft over a silicone urethral catheter (16 F) (Fig. 2).

### Patients’ follow-up

The urethral catheter is removed after 4 weeks. All patients were assessed by Q-max at 3 and 12 months post-operatively. Retrograde urethrogram and further endoscopic evaluation may be needed for patients who complained of poor flow. The safety of the procedure on erectile function was assessed by the erection domain of IIEF-15 questionnaire [17]. We defined erectile dysfunction (ED) as a decrease of 5 points or more in the postoperative IIEF score compared to the preoperative IIEF score [18]. The Clavien Dindo system was utilized to classify the post-operative complications [19]. The definition of procedural failure was an obstructive voiding pattern accompanied by cystoscopic or radiographic signs of stricture recurrence.

### Statistical analysis

The IBM SPSS software version 20.0. (Armonk, NY: IBM Corp) was utilized to analyze the data. Numbers and percentages were utilized to describe the categorical data. Range, mean, standard deviation, and median were utilized to express the quantitative data. The chi-square test was applied to compare between two groups. Conversely, for non-normally distributed quantitative data, the Mann-Whitney test was employed to compare two groups, and the Friedman test was used to determine the significance across periods using the Post Hoc Test (Dunn’s). A p-value of 0.05 or less was calculated as significant.

## Results

The median patients’ age was 42.0 and 40.0 years in groups I and II, respectively. The median BMI was 25.2 and 25.7 in groups I and II, respectively. Table 1S in the supplementary material 2 summarizes the baseline data in both groups.

The success of the procedure was comparable in both groups [55 patients (91.7%) vs. 66 patients (90.4%)] in the ventral and the dorsal groups, respectively (*p* = 0.801). Recurrence of the stricture (confirmed by imaging and endoscopy) occurred in twelve (9%) patients (five in Group I and seven in Group II). The recurrences were observed at the proximal anastomotic site in eight cases and at the distal anastomotic site in four patients. All the recurrent strictures were approximately (1 cm) and treated by endoscopic dilatation in ten patients, while the remaining two patients required a redo urethroplasty. Transient ED (at 3 month follow-up) was detected in 5% of the group I vs. 25% of the group II (*p* = 0.002). Permanent ED (at 12 month follow-up) was detected in 1.7% of the ventral group and 13.7% of the dorsal group (*p* = 0.012). The median 3 months postoperative IIEF score was 28.5 in group I vs. 25 in group II (*P* < 0.001), and the median 12 months postoperative IIEF score was 28.5 and 25 in Group I and II, respectively (*p* < 0.001) (Table 2S in supplementary material 2).

The post-operative complications were comparable in both groups (5% in Group I vs. 5.5% in Group II); wound complications (infection or hematoma) were noticed in one patient in Group I and three patients in Group II. Donor site complications were encountered in two patients in Group I and one patient in group II. All these complications were Clavein grade I and managed conservatively.

(Table 3S in supplementary material 2) compares the baseline, 3 and 12 month Q-max, and IIEF scores in both groups. There was a significant improvement in post-operative Q-max in both groups. The IIEF score (pre- and post-operative) was comparable in group I but significantly reduced postoperatively in group II.

## Discussion

As augmentation BMG urethroplasty avoids most of the sexual drawbacks of conventional urethral transection, it is regarded as the surgery of choice for long bulbar urethral strictures [20, 21]. The choice between dorsal and ventral onlay approaches depends mainly on the surgeon’s experience, the sexual activity, and the surgical history of the patient. One of the advantages of the ventral BMG bulbar urethroplasty is that it is easier, needs less urethral mobilization, and avoids dissection in the intercrural space. On the other hand, the dorsal technique for PBUS necessitates dissection of intercrural space that may cause inadvertent disruption of erectile nerves and may increase the possibility of post-urethroplasty ED [9, 22].

In the current study, we reported a similar success rate of 91.7% for the ventral technique and 90.4% for the dorsal technique (*p* = 0.801). In line with our results, Shalkamy et al. [23] reported a comparable success rate of (88.3% vs. 87.1%, *p* = 0.68) for the dorsal and the ventral techniques, respectively. Similarly, Vasudeva P et al. [12] showed that the success rate was similar between dorsal and ventral group (92.5% vs. 90%), respectively. On the other hand, Pansadoro et al. [24], retrospectively analyzed 56 dorsal and 9 ventral onlay BMG urethroplasties reporting less recurrences in the dorsal technique (2%) vs. (11%) in the ventral group; however, this difference was not significant. In contrast to our study, the number of patients in most of these studies was small to draw a firm conclusion regarding the recurrence rates equivalency. Furthermore, the DeVo randomized controlled trial that compares the dorsal onlay versus ventral onlay graft in the management of 240 patients with isolated bulbar urethral strictures is still ongoing and its final results are expected to be published in 2026 [25]. Noteworthy, Barbagli and colleagues [15], evaluated the role of VOBMGU in non-traumatic PBUS and reported a success rate of 85.5% with a mean postoperative Q-max of 23.66 ml/s (range: 13–60) which is in agreement with results of our VOBMGU technique.

However, a number of studies found that the ED incidence after various bulbar urethroplasty procedures varied, ranging from 0 to 20%, with the predominance of temporary ED following anastomotic urethroplasties [26–30]. In this study, the incidence of transient and permanent ED was significantly higher in group II. In the contrary to our results, according to Shalkamy et al. [23] there was no significant difference in the incidence of transitory and persistent ED between the dorsal and ventral procedures (3.9% vs. 1.6%; *p* = 0.41) (3.2% vs. 1.9%; *p* = 0.6), respectively. The dorsal technique results in tissue inflammation and edema, nerve roots compression, and vessels disruptions, all of which require time to heal [20]. However, despite this, the authors were unable to find a significant difference between the two approaches as regards the postoperative ED without mentioning any explanation. Indeed, their study was dealing with bulbar strictures in general and not the PBUS specifically, as we mentioned before about the difference in anatomical relations of the NVB to the proximal, middle, and distal bulbar urethra [13, 14]. This might explain the comparable rates of postoperative ED between dorsal and ventral techniques in their study [23].

Furthermore, Blakely S et al. [15] in their study conducted on 16 males who underwent dorsal graft urethroplasty in patients with membranous urethral strictures reported no change in postoperative SHIM score (median 21) compared to the preoperative score median (20.5). This paper included only 10 patients who completed the preoperative and post-operative SHIM questionnaire. Despite the removal of the fibrotic tissue in the inter-crural area, the authors found no effect on erection in their sexually potent patients, which they ascribed to their modified approach that circumvents circumferential urethral dissection and transection [15]. However, one of the major drawbacks of their study is the small sample size, which affects the reliability of their results.

Concerning the operative time (OT) in this study, the median OT was significantly shorter in ventral group (105 min vs. 110 min in the dorsal group, *p* = 0.037). This observation could be attributed to the ease and rapid access to the urethra ventrally without any dissection related to the urethra, and hence the rapid urethrotomy and quilting of the BMG, followed by the closure of the urethra over the graft. Similarly, Shalkamy et al. [23] reported that the mean OT was significantly shorter in the ventral group compared to the dorsal group (130.2 versus 146.1 min, p **<** 0.001).

Noteworthy, the post-operative complications in the current study were comparable in both groups (5% in the ventral group vs. 5.5% in the dorsal group). This was similar to the results of Shalkamy et al. [23], who found that the postoperative complications were comparable among both groups (15.5% in the dorsal group vs. 19.4% in the ventral group. *P* = 0.53). Vasudeva P et al. [9] mentioned in their study that 7.5% in the dorsal group and 15% in the ventral group developed postoperative complications in the form of wound and donor site complications.

Regarding the outcome of bulbar urethroplasty, recent guidelines stated that due to the limited and conflicting evidence and for preservation of erectile function, no recommendation can be made about the routine use of nerve and muscle-sparing modification, while the location of the graft has no impact on patency rates and guidelines recommend that the use of different surgical techniques described with ventral, lateral, dorsolateral, or dorsal graft as an onlay or an inlay should be done according to surgical practice, expertise, and intra-operative findings [31]. Thus, we believe that our study might provide a new insight to the advantages of the VOBMGU technique regarding the better erectile function results without compromising the patency rates; however, we recommend that high-quality RCTs are still needed to validate our results.

Our study is not devoid of limitations. It is a retrospective study; thus, the risk of selection bias cannot be excluded and this might confound the comparison. The relatively small number of included patients (*n* = 133). Despite the number is considered larger than several published articles comparing both techniques, however, this may reduce the generalizability of the findings, and the power to detect small differences when dividing this number into two groups. The functional outcome was evaluated at short term follow-up (12 months). On the other hand, our study has several points of strength. First of all, this is the first study in the literature focusing specifically on the treatment of PBUS in sexually active men and compares the erectile functions in both ventral and dorsal approaches. All surgeries are performed by a single expert surgeon to avoid heterogenicity of the outcomes if multiple surgeons were involved. Further prospective randomized controlled studies are warranted to validate our results.

## Conclusion

Both VOBMGU and DOBMGU are effective in treating PBUS with comparable urinary outcomes. The ventral approach is significantly associated with a low incidence of transient and permanent postoperative erectile dysfunction when compared to the dorsal technique. Therefore, in sexually active men, VOBMGU may be the future treatment for PBUS, however, further RCTs are warranted to validate our results and longer follow-up is essential to fully assess the durability of the outcomes and the long-term impact on erectile function.

## Electronic supplementary material

Below is the link to the electronic supplementary material.

Supplementary Material 1

Supplementary Material 2

## Data Availability

The data that support the findings of this study are available from the corresponding author upon reasonable request.
