# Supplementary material for: Ventral versus dorsal onlay buccal mucosal graft urethroplasty for non-traumatic proximal bulbar urethral strictures in sexually active men: erectile and urinary functions
Source: World J Urol. 2025 Jan 27;43(1):87. doi: 10.1007/s00345-025-05441-7 (PMC11772550; doi:10.1007/s00345-025-05441-7)
Supplement: Supplementary file 1 — Supplementary Material 1 [file 345_2025_5441_MOESM1_ESM.docx]

**Supplementary material**

**
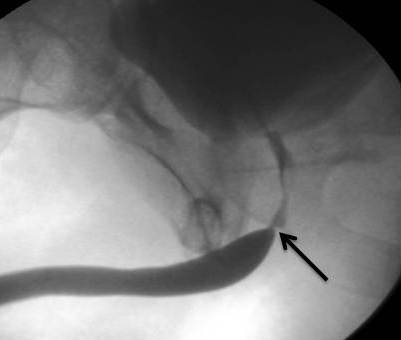
**

**Fig. 1S** Retrograde urethrogram (RUG) shows proximal bulbar urethral stricture about 1 cm.


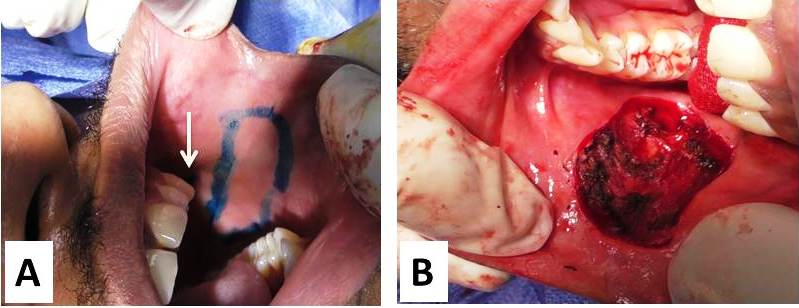


**Fig. 2S** BMG harvest under local anaesthesia**. A** Marking the boundaries of the buccal graft (*the white arrow* points to the Stenson's duct). **B** The bed of the graft after proper hemostasis.


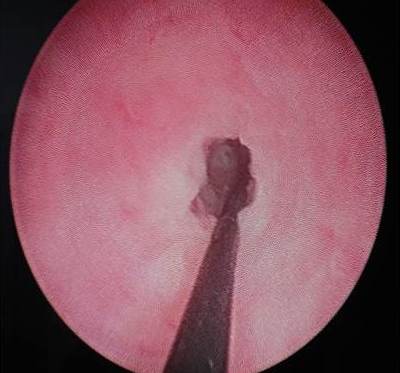


**Fig. 3S** Endoscopic view shows a tight bulbar urethral stricture and a Terumo guide wire is inserted into the urinary bladder.
