# Supplementary material for: Ventral versus dorsal onlay buccal mucosal graft urethroplasty for non-traumatic proximal bulbar urethral strictures in sexually active men: erectile and urinary functions
Source: World J Urol. 2025 Jan 27;43(1):87. doi: 10.1007/s00345-025-05441-7 (PMC11772550; doi:10.1007/s00345-025-05441-7)
Supplement: Supplementary file 2 — Supplementary Material 2 [file 345_2025_5441_MOESM2_ESM.docx]

**Table 1S** Baseline data in both groups.

| **Baseline data** | **Ventral group (n = 60)** | **Dorsal group (n = 73)** | **Test of Sig.** | **p** |
| --- | --- | --- | --- | --- |
| **Age at time of surgery** |  |  |  |  |
| Mean ± SD. | 39.5 ± 9.2 | 38.8 ± 8.5 | U= 2056.50 | 0.545 |
| Median (Min. – Max.) | 42.0 (22.0 – 52.0) | 40.0 (23.0 – 52.0) |  |  |
| **BMI (kg/m^2^)** |  |  |  |  |
| Mean ± SD. | 25.8 ± 7.1 | 25.3 ± 6.6 | U= 2120.0 | 0.751 |
| Median (Min. – Max.) | 25.2 (13.0 – 55.0) | 25.7 (13.0 – 55.0) |  |  |
| **Smoker** | 6(10%) | 6(8.2%) | χ^2^= 0.127 | 0.721 |
| **Diabetes** | 5(8.3%) | 7(9.6%) | χ^2^= 0.063 | 0.801 |
| **Main symptoms** |  |  |  |  |
| LUTs | 18(30%) | 23(31.5%) | χ^2^= 3.258 | 0.516 |
| UTI | 16(26.7%) | 16(21.9%) |  |  |
| Hematuria | 5(8.3%) | 12(16.4%) |  |  |
| Poor flow | 16(26.7%) | 13(17.8%) |  |  |
| Genital pain | 5(8.3%) | 9(12.3%) |  |  |
| **Q-max pre-operative** |  |  |  |  |
| Mean ± SD. | 6.3 ± 2.7 | 6.4 ± 2.5 | U= 2143.0 | 0.830 |
| Median (Min. – Max.) | 6.0 (2.0 – 11.0) | 6.0 (2.0 – 12.0) |  |  |
| **Stricture length (cm)** |  |  |  |  |
| Mean ± SD. | 2.3 ± 0.5 | 2.3 ± 0.5 | U= 2110.0 | 0.708 |
| Median (Min. – Max.) | 2.5 (1.5 – 3) | 2 (1.5 – 3.2) |  |  |
| **IIEF score pre-operative** |  |  |  |  |
| Mean ± SD. | 28.5 ± 1.1 | 28.2 ± 1.2 | U= 1950.0 | 0.264 |
| Median (Min. – Max.) | 28.5 (27.0 – 30.0) | 28.0 (26.0 – 30.0) |  |  |

SD: **Standard deviation U: Mann Whitney test**

**χ^2^: Chi square test FET: Fisher Exact test**

p: p value for comparing between the two studied groups

*: Statistically significant at p ≤ 0.05

**Table 2S** Operative and postoperative data in both groups**.**

| **data** | **Ventral group (n = 60)** | **Dorsal group (n = 73)** | **Test of Sig.** | **p** |
| --- | --- | --- | --- | --- |
| **Stricture etiology** |  |  |  |  |
| Idiopathic | 30(50%) | 31(42.5%) | χ^2^= 2.195 | 0.334 |
| Catheter induced | 15(25%) | 27(37%) |  |  |
| Inflammatory | 15(25%) | 15(20.5%) |  |  |
| **Previous treatment** |  |  |  |  |
| No | 45(75%) | 54(74%) | χ^2^= 0.018 | 0.892 |
| VIU | 15(25%) | 19(26%) |  |  |
| **Number of urethrotomies** |  |  |  |  |
| Mean ± SD. | 2.6 ± 0.9 | 2.4 ± 0.9 | U= 115.0 | 0.529 |
| Median (Min. – Max.) | 3 (1 – 4) | 2 (1 – 4) |  |  |
| **Graft length (cm)** |  |  |  |  |
| Mean ± SD. | 4.1 ± 0.8 | 4.3 ± 0.7 | U= 1815.0 | 0.071 |
| Median (Min. – Max.) | 4 (2.5 – 6) | 4 (3 – 6) |  |  |
| **Operative time** |  |  |  |  |
| Mean ± SD. | 106.4 ± 11 | 110.6 ± 11.4 | t= 2.109^*^ | 0.037^*^ |
| Median (Min. – Max.) | 105 (90 – 130) | 110 (95 – 135) |  |  |
| **Success** | 55(91.7%) | 66(90.4%) | χ^2^= 0.063 | 0.801 |
| **Transient ED** | 3(5%) | 18(24.7%) | χ^2^= 9.571^*^ | 0.002^*^ |
| **Permanent ED** | 1(1.7%) | 10(13.7%) | χ^2^=6.284 | ^FE^p=0.012^*^ |
| **Q-max** |  |  |  |  |
| **Post-operative 3 months** |  |  |  |  |
| Mean ± SD. | 30.3 ± 9.1 | 30.9 ± 8.3 | U= 2139.50 | 0.819 |
| Median (Min. – Max.) | 33 (6 – 44) | 33 (5 – 44) |  |  |
| **Post-operative 12 months** |  |  |  |  |
| Mean ± SD. | 28 ± 8.2 | 28.2 ± 8.1 | U= 2077.0 | 0.608 |
| Median (Min. – Max.) | 29 (5 – 44) | 30 (4 – 44) |  |  |
| **IIEF score** |  |  |  |  |
| **Post-operative (3 months)** |  |  |  |  |
| Mean ± SD. | 28.2 ± 2 | 22.1 ± 5.8 | U= 215.50^*^ | <0.001^*^ |
| Median (Min. – Max.) | 28.5 (20 – 30) | 25 (8 – 28) |  |  |
| **Post-operative (12 months)** |  |  |  |  |
| Mean ± SD. | 28.4 ± 1.6 | 24.4 ± 3.9 | U= 334.0^*^ | <0.001^*^ |
| Median (Min. – Max.) | 28.5 (20 – 30) | 25 (10 – 28) |  |  |

SD: **Standard deviation U: Mann Whitney test**  **t: Student t-test**

**χ^2^: Chi square test FET: Fisher Exact test ED: erectile dysfunction**

p: p value for comparing between the two studied groups

*: Statistically significant at p ≤ 0.05

**Table 3S** Comparison between the pre-operative, 3 and 12 months Q-max and IIEF score in both groups.

|  |  | **Pre-operative** | **Post-operative 3 months** | **Post-operative 12 months** | **Fr** | **p** |
| --- | --- | --- | --- | --- | --- | --- |
| **Q max** | **Ventral (n = 60)** |  |  |  |  |  |
|  | Mean ± SD. | 6.3 ± 2.7 | 30.3 ± 9.1 | 28 ± 8.2 | 87.280^*^ | <0.001^*^ |
|  | Median (Min. – Max.) | 6 (2 – 11) | 33 (6 – 44) | 29 (5 – 44) |  |  |
|  | **Sig. bet. period** | p_1_<0.001^*^,p_2_<0.001^*^,p_3_=0.075 | | |  |  |
|  | **Dorsal (n = 73)** |  |  |  |  |  |
|  | Mean ± SD. | 6.4 ± 2.5 | 30.9 ± 8.3 | 28.2 ± 8.1 | 106.443^*^ | <0.001^*^ |
|  | Median (Min. – Max.) | 6.0 (2.0 – 12.0) | 33 (5 – 44) | 30 (4 – 44) |  |  |
|  | **Sig. bet. period** | p_1_<0.001^*^,p_2_<0.001^*^,p_3_=0.012^*^ | | |  |  |
| **IIEF** | **Ventral (n = 60)** |  |  |  |  |  |
|  | Mean ± SD. | 28.5 ± 1.1 | 28.2 ± 2 | 28.4 ± 1.6 | 0.043 | 0.979 |
|  | Median (Min. – Max.) | 28.5 (27 – 30) | 28.5 (20 – 30) | 28.5 (20 – 30) |  |  |
|  | **Dorsal (n = 73)** |  |  |  |  |  |
|  | Mean ± SD. | 28.2 ± 1.2 | 22.1 ± 5.8 | 24.4 ± 3.9 | 116.312^*^ | <0.001^*^ |
|  | Median (Min. – Max.) | 28.0 (26.0 – 30.0) | 25 (8 – 28) | 25 (10 – 28) |  |  |
|  | **Sig. bet. period** | p_1_<0.001^*^,p_2_<0.001^*^,p_3_=0.001^*^ | | |  |  |

SD: **Standard deviation**

**Fr**: **Friedman test**, Sig. bet. periods was done using **Post Hoc Test** (**Dunn's)**

p: p value for comparing between the three studied periods

p_1_: p value for comparing between **pre-operative** and **Post-operative 3 months**

p_2_: p value for comparing between **pre-operative** and **Post-operative 12 months**

p_3_: p value for comparing between **Post-operative 3 months** and **Post-operative 12 months**

*: Statistically significant at p ≤ 0.05
